# Insulin/IGF-1R, SIRT1, and FOXOs Pathways—An Intriguing Interaction Platform for Bone and Osteosarcoma

**DOI:** 10.3389/fendo.2019.00093

**Published:** 2019-03-01

**Authors:** Consolato Sergi, Fan Shen, Song-Mei Liu

**Affiliations:** ^1^Department of Orthopedics, Tianyou Hospital, Wuhan University of Science and Technology, Wuhan, China; ^2^Department of Laboratory Medicine and Pathology, University of Alberta, Edmonton, AB, Canada; ^3^Department of Pediatrics, Stollery Children's Hospital, Edmonton, AB, Canada; ^4^Center for Gene Diagnosis, Zhongnan Hospital of Wuhan University, Wuhan, China

**Keywords:** IGF1, FOXO, SIRT1, signaling pathways, bone, osteosarcoma

## Abstract

Aging is a substantial risk factor for the development of osteoarthritis (OA) and, probably, an essential substrate for the development of neoplastic disease of the bone, such as osteosarcoma, which is the most common malignant mesenchymal primary bone tumor. Genetic studies have established that the insulin/insulin-like growth factor 1 (IGF-1)/phosphatidylinositol-3 kinase (PI3K)/AKT (Protein Kinase B) signal transduction pathway is involved across species, including nematodes, fruit flies, and mammals. SIRT1, a phylogenetically-conserved family of deacetylases, seems to play pleiotropic effects in epithelial malignancies of the liver and interact with the IGF-1/PI3K/AKT signal transduction pathway. Some of the most critical processes in degenerative conditions may indeed include the insulin/IGF1R and SIRT1 signaling pathways as well as some specific transcription factors. The Forkhead box O (FOXO) transcription factors (FOXOs) control diverse cellular functions, such as metabolism, longevity, and cell death. FOXOs play a critical role in the IGF-1/PI3K/AKT signal transduction pathway. FOXOs can indeed be modulated to reduce age-related diseases. FOXOs have advantageous inhibitory effects on fibroblast and myofibroblast activation, which are accompanied by a subsequent excessive production of extracellular matrix. FOXOs can block or decrease the fibrosis levels in numerous organs. Previously, we observed a correlation between nuclear FOXO3 and high caspase-8 expression, which induces cellular apoptosis in response to harmful external stimuli. In this perspective, we emphasize the current advances and interactions involving the insulin/IGF1R, SIRT1, and FOXOs pathways in the bone and osteosarcoma for a better understanding of the mechanisms potentially underpinning tissue degeneration and tumorigenesis.

## Introduction

Aging is a substantial risk factor for the development of inflammatory conditions, such as osteoarthritis (OA) and, probably, other degenerative, and neoplastic diseases of the bone, such as Paget disease of the bone and osteosarcoma (1, 2). Some growth factors linked to cartilage repair following damage in animal models have been considered to increase the risk of neoplasia (3–7). In this paper, we review the insulin/IGF1R, SIRT1, and FOXOs signaling pathways and emphasize the interaction involving these crucial factors in the bone physiology and oncogenesis, with regard to osteosarcoma, which is considered the most common mesenchymal malignant primary tumor of the skeletal system.

## Insulin/IGF1R Signaling Pathway

The insulin/IGF-1 signaling system (IIS) is the route that regulates not only the organism's metabolism, but also the growth, development, and longevity concerning the availability status of nutrients. It is an ancient system. In fact, it is highly conserved across species. In invertebrates, e.g., in the worm *C. elegans*, the IIS system begins with the secretion of numerous, insulin-like peptides in reply to food or, ultimately, to the sensory perception of food. These insulin-like ligands can connect to a single (common) receptor, called the insulin/IGF-1 like tyrosine kinase receptor or *dauer* formation 2 (DAF-2). In the 1990s, two DAF genes, DAF-2, and DAF-16, were discovered after isolating *dauer-constitutive* (DAF-c) mutants and *dauer-defective* mutants (DAF-d). The worm *C. elegans*, under conditions of high population density and low food, forms an alternative 3rd larval stage, called the *dauer* stage. This stage is resistant to dehydration and harsh environments (8). The *C. elegans* genome encodes AGE-1 adaptor protein (AAP-1), a single PI3K adaptor subunit, and a putative IRS homolog, i.e., the adaptor protein or insulin receptor substrate (IST-1) homolog (9). After the ligand binds, the signal is progressively transduced from the activated receptor to AGE-1, which is a phosphatidylinositol 3-kinase either directly or using the adaptor protein called IST-1 (9). The phosphatidylinositol 3-kinase AGE-1 changes the phospholipid PIP2 into the second messenger PIP3. Subsequently, the increased level of PIP3 initiates the 3-phosphoinositide-dependent protein kinase 1 (PDK1) and the protein kinases B1 and B2 (PKB1 and PKB2). Ultimately, it leads to the phosphorylation of the DAF-16 molecule, which causes its extrusion from the nucleus to the cytoplasm (10). DAF-18, a homolog of the mammalian phosphatase and tensin homolog (PTEN), can dephosphorylate PIP3 to PIP2. Gene mutations in daf-2 and kinase components of the IIS pathway harboring reduction of functional significance can extend the life span of the worm. Conversely, mutations harboring the same meaning but in daf-18 abolish the life-span extension of daf-2 and age-1 mutants. The downstream targets of DAF-16 include metabolic genes, cellular stress response genes, and genes encoding antimicrobial peptides (11, 12). The fruit fly (*Drosophila melanogaster*) shows powerful similarities to *C. elegans* about the IIS pathway. In the fruit fly, multiple extracellular ligands are binding to a single tyrosine kinase receptor, which is a transmembrane protein, the insulin/IGF-1 common receptor. The binding of the ligands to the common receptor promotes some intracellular phosphorylation events that end in the phosphorylation and nuclear extrusion of dFOXO. In the fruit fly, several indirect losses of function gene mutations have been linked to an enhancement of the life span, such as the insulin receptor and its substrate. These events are particularly pronounced in the female fruit fly. In mammals, the core of the insulin/IGF-1 signaling path is preserved, but there is an increase in complexity moving from invertebrates to vertebrates. Specifically, there are three different ligand molecules of insulin/IFG-1 receptor in mammals. They include insulin, IGF-1, and IGF-2. Also, there are three diverse mammalian insulin/IGF tyrosine kinase receptors, including insulin receptor (IR), IGF-1 receptor (IGF-1R), and the so-called orphan IR related receptor (IRR). An orphan receptor is a protein that harbors a structure similar to other identified receptors but whose endogenous ligand has not yet been discovered. Following the ligand binding, the activated IGF-1 or insulin receptor starts the phosphorylation of numerous intracellular substrates. The phosphorylated substrates give precise docking sites for intracellular effectors. These sites include the growth-factor-receptor-bound protein-2 (Grb2) and the p85 regulatory subunit of PI-3K. Eventually, it leads to the activation of two major signaling pathways, which are the Ras-MAPK pathway and the PI-3K-PKB/AKT pathway. The former path (PI-3K-PKB/AKT) has been shown to regulate most of the metabolic effects of insulin/IGF-1 signaling (13). The latter pathway (Ras-MAPK) gave evidence of the regulation of most of the effects (mitogenic) of insulin/IGF-1 signaling. Also, most of the crucial components of the insulin/IGF-1 signaling cascade show some further complexity in mammals, because different forms have been revealed that are encoded by several genes and/or isoforms determined by a single gene.

## SIRT1 Signaling Pathway

In mammals, Sirtuins constitute a family of NAD+-dependent deacetylases (14–19). Seven members (SIRT1–SIRT7) are included in this family. All of them share the conserved Sirtuin domain conferring NAD+-dependent deacetylase activity (20). However, they also have different amino- and carboxy-terminal extensions. Also, they display distinct subcellular localization and biological functions. Although SIRT1 is predominantly located in the nucleus, it transfers between the nucleus and the cytoplasm during the ontogenesis and in response to physiological stress and pathological conditions. If SIRT1 is located in the nucleus, different is the situation for SIRT2, SIRT3, SIRT4, and SIRT5. The proteins SIRT3 through SIRT5 are located in the mitochondria, while SIRT2 is mostly identified in the cytoplasm. Like SIRT1, SIRT6, and SIRT7 are localized in the nucleus. The former is a chromatin-associated protein, while the latter is found in the nucleolus. In mammals, deacetylase activity was reported as conserved, but the acyl group preference is different according to the Sirtuins with SIRT4-7 harboring a weak or, probably, undetectable deacetylase activity *in vitro* (21–23). An efficient demyristoylase activity is found in SIRT2, while SIRT5 has a demalonylase and lysine desuccinylase activities associated with an adequate and efficient NAD+-dependent protein. SIRT4 and SIRT6 harbor ADP-ribosyltransferase activity. In mammals, Sirtuins are crucial in regulating a broad variety of cellular processes, including metabolism, oxidative/anti-oxidative balance, mitochondrial homeostasis, autophagy, and apoptosis as well as pathological conditions, such as inflammation. SIRT1 has been proved to repress inflammation in multiple cells and tissues (24–26). Moreover, there is an important contribution of Sirtuins in aging and aging-related diseases, such as obesity, type 2 diabetes mellitus (T2DM), cardiovascular disease, neurodegenerative diseases, and cancer (16, 17). Most probably, SIRT1 (and SIRT6 for certain aspects) is the most extensively characterized proteins of this class. In several species and across species, the nuclear factor kappa-light-chain-enhancer of activated B cells (NF-κB) remains a chief transcriptional factor in cellular physiology. NF-κB is a complex that panels the transcription of DNA, production of several cytokines, and, ultimately, cell survival. In the cell, NF-κB is tangled in responses to stimuli such as stress, free radicals, cytokines, but also bacterial or viral antigens as well as heavy metals, ultraviolet irradiation, oxidized lipoproteins. NF-κB mediates the expression of multiple inflammatory factors, including IL-1β, IL-6, and TNF-α. The nuclear translocation and activation of NF-κB rely on its acetylation. SIRT1 deacetylates NF-κB (p65 subunit) at lysine 310 (K310). It inhibits the transcriptional activity of NF-κB. A co-repressive action has also been identified with transducing-like enhancer of split 1 (TLE1). SIRT1 interacts with TLE1 to inhibit NF-κB-mediated transcription and suppress inflammation (27, 28). Other two actions of the SIRT1 are well-known and include (1) the deacetylation of activator protein-1 (AP-1) able to decrease the expression of cyclooxygenase 2 (COX-2) in macrophages and (2) the deacetylation of TP53 to repress macrophage activation. Sirtuins significantly contribute to their functions on insulin resistance. The activation of SIRT1 leads to the repression of c-Jun N-terminal kinases (JNKs) and inhibitor of NF-κB kinase subunit beta (IKK-β) inflammatory pathways substantially. JNKs were initially discovered as kinases that bind and phosphorylate c-Jun on Ser-63 and Ser-73 (two serine amino acid residues) within its transcriptional activation domain (29, 30). They are a member of the mitogen-activated protein kinase (MAPK) family and are responsive to stress stimuli. IKK- β is a protein that in humans is encoded by the gene labeled inhibitor of kappa light polypeptide and gene enhancer in B-cells, kinase beta (IKBKB). SIRT1 improves glucose tolerance, reduced hyperinsulinemia, and enhanced systemic insulin sensitivity and Sirt1 controls specifically the inflammatory status of macrophages and T lymphocytes regulating the metabolism and that of adipose tissues in obese mice (31, 32). Besides, SIRT1 promotes mitochondrial biogenesis by deacetylating the peroxisome proliferator-activated receptor and gamma coactivator 1-alpha (PGC-1α), which is a transcriptional coactivator that regulates mitochondrial biogenesis and respiration. SIRT1 activator resveratrol induces PGC-1α increasing the number of mitochondria, and this may have a beneficial effect in T2DM patients, who have fewer mitochondria in the muscle than insulin-sensitive individuals. SIRT1 activator resveratrol inducing PGC-1α activity protects mice against diet-induced obesity and insulin resistance (33–35). Mitochondria biogenesis is found in a balance with the clearance of damaged mitochondria (mitophagy or mitochondria autophagy). SIRT1 binds to and deacetylates autophagy (ATG) regulators (e.g., ATG5, ATG7, and ATG8) to promote mitophagy (29, 36–39). SIRT1 also deacetylates Forkhead box protein O1 (FoxO1) and Forkhead box protein O3a (FoxO3a). Both deacetylations induce the expression of elements of the autophagy machinery. The deacetylation and activation of FoxO3a upregulate the expression of MnSOD and catalase, which is an enzyme detected in almost all living organisms exposed to O_2_. Catalase catalyzes the decomposition of hydrogen peroxide (H_2_O_2_) to water and O_2_. Calatase plays a major role in protecting the cell from oxidative damage by reactive oxygen species (ROS). Finally, SIRT1 promotes the transcriptional activity of Nuclear factor (erythroid-derived 2)-like 2 (NRF2). SIRT1 acts in this way by deacetylating it. SIRT1 upregulates the expression of NRF2 target antioxidant genes, including mitochondrial antioxidant manganese superoxide dismutase (MnSOD), catalase, heme oxygenase-1 (HO-1), and glutathione (29, 40, 41). Overall, Sirtuins are histone deacetylases that are crucial in regulating organismal lifespan as well as oxidative stress and DNA damage.

## FOXOs Signaling Pathway—General Remarks

An impressive class of transcription factors for cancer therapeutic modulation is represented by the forkhead box transcription factors (FOXO) family. FOXOs or FOXO proteins are growth factors and stress-related factors. These transcription factors naturally reside in the nucleus of cells and function as regulators of gene transcription. FOXO proteins may be transferred to the cytoplasm and go through degradation of the ubiquitin-proteasome pathway (UPP), which is the chief mechanism for protein catabolism in the mammalian cell. Following translation of the mRNA into proteins, and no cellular survival initiative of growth factors, FOXOs translocate to the nucleus upregulating a series of target genes of the cell cycle, stress resistance, and longevity. FOXOs regulate numerous cellular functions, and these functions include cellular differentiation, cellular proliferation, DNA damage, apoptosis, DNA repair, and oxidative stress modulation (42–45). These very delicate functions indicate how dysregulation of the FOXOs may implicate abnormal cellular and tissue physiology, tumorigenesis, and neoplastic progression. Different from most common transcription regulators, such as extracellular signal-regulated kinase (ERK), which are located in the cytoplasm where kinases are phosphorylated and translocated into the nucleus, FOXOs are transcription factors with a nuclear location. Growth factor pathways endorse the nuclear exclusion and translocation of phosphorylated FOXO to the cytoplasm. In the cytosol, the phosphorylated FOXO is subjected to degradation via the UPP. The founding member of the FOX family is the forkhead transcription factor of the forkhead box (FOX) family in the fruit fly *Drosophila melanogaster*, of which a mutation in Foxo genes results in defective head shrinkage. The forkhead box is a conserved domain, which has been described due to a butterfly-like appearance on nuclear magnetic resonance and X-ray crystallography. This domain consists of three α-helices and three β-sheets that are accompanied with two loops usually referred to like the wings. In invertebrates, there is only one FOXO gene, termed dFOXO in the fruit fly and daf-16 in the worm. Conversely, there are four FOXO genes, FOXO1, 3, 4, and 6 in mammals. In the mammals, forkhead transcription factors of the O class, i.e., FOXO1, FOXO3, FOXO4, and FOXO6 proteins, play a paramount role in the usual functional complexity of the cell, its proliferation, differentiation, and death as well as in the progression of several diseases. FOXO1 is a transcription factor that plays crucial roles in the regulation of gluconeogenesis and glycogenolysis by insulin signaling and is also vital to the decision for a preadipocyte to commit to adipogenesis. In mammals, FOXO localization at the subcellular level is exquisitely controlled by post-translational modifications, including ubiquitination, phosphorylation, and acetylation (29, 44–52). During development and tumorigenesis, the protein kinase B (AKT) path plays a significant role in cell growth and survival. AKT is activated by AKT kinase PDK1/2. AKT kinase PDK1/2 is considered to be a downstream target kinase of phospholipid kinase phosphatidylinositol 3-kinase (PI3K). In mammals, there are three isoforms which are determined by distinct loci. FOXOs are proteins active in growth factor signaling. In the pathway, they are positioned downstream of AKT. In addition to AKT, there are negative regulators of FOXOs, including the casein kinase 1 (CK1), dual-specificity tyrosine-phosphorylation-regulated kinase 1A (DYRK1A), serum and glucocorticoid-regulated kinase (SGK), IκB kinase (IKK), and the ubiquitous ERK. Other kinases, which control FOXOs are c-Jun N-terminal kinase (JNK) and mammalian ste20-like kinase (MST1) which act under circumstances of elevated oxidative stress. In the last few years, it has been suggested how FOXO regulation may contribute to fibrosis of several organs (42). In particular, FOXO1/3 have been demonstrated to have promising inhibitory effects on fibroblast activation and extracellular matrix production improving the degree of fibrosis levels in several organs, including the heart, kidney, liver, and lung (42). FOXOs have been cataloged to be tumor suppressors due to their antiproliferative and pro-apoptotic actions, despite some data revealed unpredicted functions of FOXOs in the advancement of cancer and in modifying responses to cancer treatment (49). A complex array of posttranslational modifications regulates FOXO transcriptional activity. These posttranslational modifications can be either activating or inactivating. These modifications alter modify the DNA binding affinity, nuclear import and export, and alter the pattern of transcriptional activity for specific target (53). FOXO factors play a crucial role in cell fate decision.

## FOXO and Bone

In a concerted fashion, bone is continuously degraded and replaced by the action of operating cells toward growth and operating cells toward remodeling. The builder cells are the osteoblasts, while the refining cells are the osteoclasts. This process of regeneration is active throughout the entire life of a vertebrate organism and is also present during tumorigenesis. Neoplasm is intrinsically linked to inflammation, and this aspect is not only relevant to the liver, where parasitic cholangitis can evolve to cancer, but it is a phenomenon across several organs and structures, including the skeletal system (54). Osteoclasts, which are short-lived giant multinucleated cells, arise from the fusion of myeloid lineage progenitor cells. They are under the influence of macrophage colony-stimulating factor (M-CSF) and the receptor activator of nuclear κ-B ligand (RANKL) and their respective receptors (55). The action of proteolytic enzymes and hydrogen ions (H^+^) on the mineralized bone matrix evolves along all life of an individual. The “podosome belt” that tightly adheres to the bone area targeted for removal relies on a polarized secretion of proteolytic enzymes and H^+^ ions (56–58). In the skeleton of an adult individual, bone mass is maintained, but the process is more complicated than thought initially. The maintenance of the bone mass requires the activity of both the re-absorption process and the re-apposition process. Thus, the resorption process is regulated by the osteoclast, while the deposition of new bone relies on osteoblasts. Human pathology and aging determine an imbalance between bone deposition and resorption. Conversely, osteoblasts are short-lived mesenchymal cells derived from bone progenitor cells that express the osteoblast identifying transcriptions factors RUNX2 and OSTERIX1 (59–62). FOXOs repress proliferation of OSTERIX1+ committed osteoblast precursors by inhibiting the canonical Wnt/β-catenin signaling (59). The osteoblasts are responsible for the deposition of osseous matrix (osteoid) in the bony tissue spaces emptied by the osteoclasts. One of the critical aspects of bone remodeling is played by the Wnt signaling (63). The WNT proteins present in the extracellular space bind to receptor frizzled and coreceptor lipoprotein-like receptor protein (LRP) 5/6 present on the cell surface of mesenchymal progenitors. The Wnt-frizzled and Wnt-LRP 5/6 binding triggers an intracellular set of events, which culminates in the release of β-catenin from a proteasomal degradation complex, its translocation into the nucleus, and subsequent binding T-cell factor/lymphoid enhancer factor (TCF/LEF) transcription factors. This bond β-catenin—TCF/LEF can activate or suppress the expression of Wnt target genes (64). Although most of the osteoblasts once finished their function die by apoptosis, a subgroup of them will incorporate in the bone matrix becoming long-lived dendritic cells called osteocytes. The osteocytes are a strong pillar in bone remodeling by producing RANKL and sclerostin, which are essential for both bone resorption and deposition, respectively (65).

**Figure 1:**
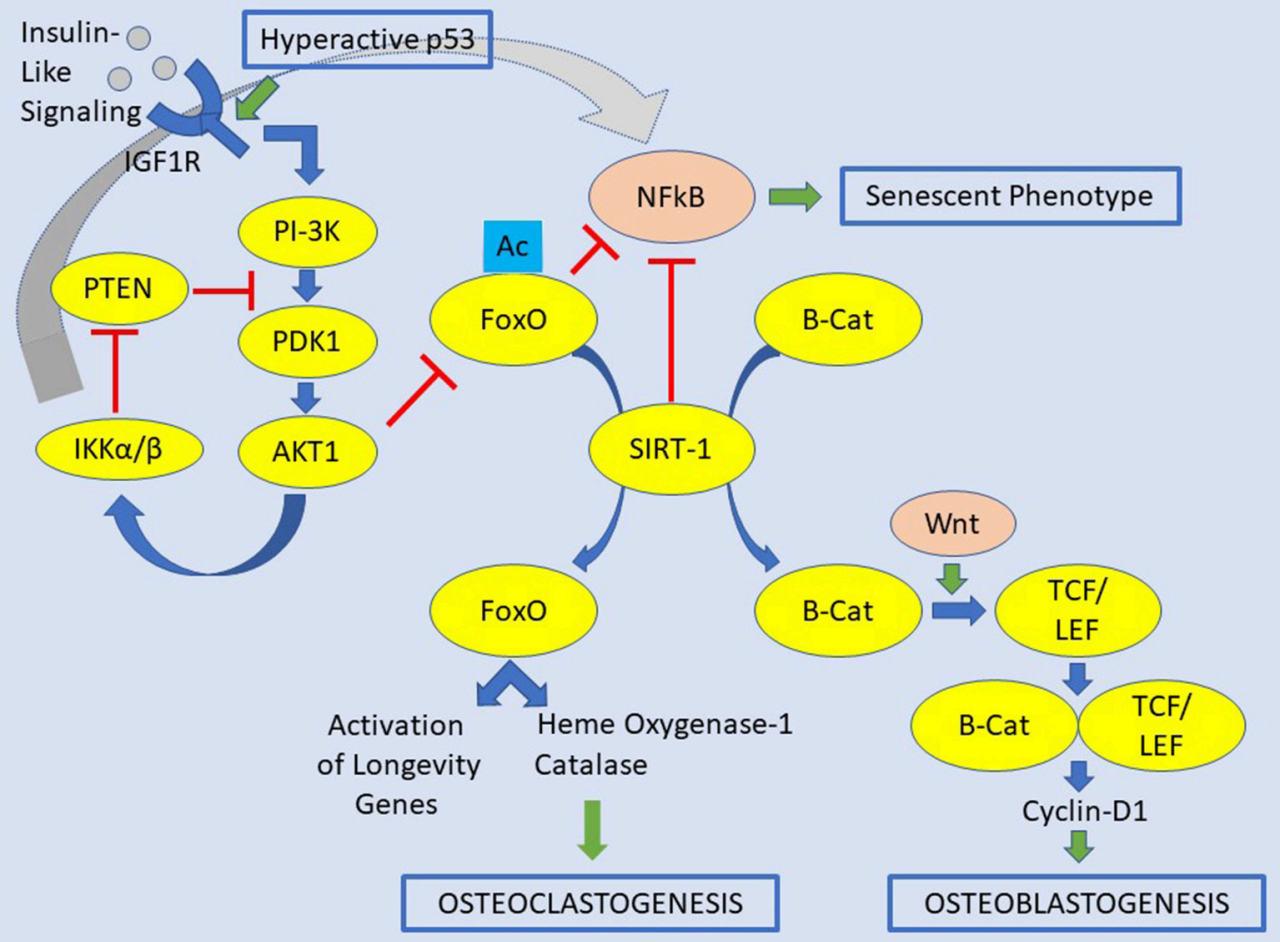
Signaling pathways contemplating insulin-like signaling/IGFR1, FOXOs, and SIRT1 for the osteoclastogenesis and osteoblastogenesis. In this original picture is also depicted the occurrence of a hyperactive p53. A hyperactive p53 would be a condition leading to osteosarcoma-genesis. The abbreviations of this figure are shown in the text and please see the text for details.

The FOXO1, 3, and 4 proteins are critical for bone development, and the control of bone mass in both physiology and pathology (Figure 1). FOXOs are essential regulators of osteoclast differentiation and bone resorption by decreasing the ROS (66). The loss of all FOXO1, 3, and 4 in osteoclast progenitors does increase proliferation, osteoclast formation, and bone resorption, which lead to reduced trabecular and cortical bone mass. Contrarywise, gain-of-function of FOXO3 inhibits bone resorption by osteoclast differentiation. This aspect results by an increase of the expression of catalase, as an example of antioxidant enzymes able to impede H_2_O_2_ (66). The accumulation of ROS is due to RANKL, which decrease the levels and activity of FOXO1, 3, and 4 via AKT-mediated phosphorylation and proteasomal degradation (66–69). FOXOs also excite heme oxygenase-1 (HO-1) to be expressed in osteoclast progenitors. Furthermore, macrophages are the site where HO-1 catabolizes heme and attenuates oxidative phosphorylation and ATP production in mitochondria (70). Overall, HO-1 contributes to the anti-osteoclastogenic effects of FOXOs. Further, FOXOs act directly to reduce the osteoblastogenesis by restraining Wnt signaling in which FOXOs bind to and sidetrack β-catenin away from TCF/LEF-mediated transcription. Consequently, there is a decrease in cyclin D1 and cell proliferation, and finally bone mass (59). However, FOXOs seems also to promote survival of osteoblasts by increasing the expression of catalase and superoxide dismutase, two major antioxidant enzymes, preventing oxidative cellular stress (71, 72). Also, FOXO1 promotes the accumulation of glutathione, a peptide that reduces ROS due to its redox-active sulfhydryl moieties. There is also a paracrine mechanism, but details of this process are still missing. The anti-osteoclastogenic actions of FOXOs are probably due to stimulation of osteoprotegerin (OPG), which is the decoy receptor for RANKL (59, 73).

## Molecular Interaction Between IGF-1, SIRT1, and FOXOs

The PI3K-PKB/AKT pathway is the canonical pathway regulating the transcriptional activity of FOXOs. While FOXOs and SIRT1 add conceptually to the longevity of the bone through an equilibrated balance of bone formation and bone remodeling, the action related to IGF1 and IGF-R1 may act in the opposite direction. SIRT1 mediates posttranslational modifications of FOXOs and seems to prevent bone resorption and stimulate bone formation. Multiple kinases can modulate FOXOs through phosphorylation, and post-translational changes may work influencing FOXO activity. These modifications include methylation, ubiquitination, acetylation, PARylation, glycosylation, and hydroxylation. Poly ADP-ribosylation (PARylation) is a highly dynamic post-translation protein modification at DNA lesions, which is catalyzed by poly (ADP-ribose) polymerases (74). The accumulation of FOXOs in the nucleus determines its binding to various transcription-cofactors regulating the transcription of genes involved in the cell cycle, apoptosis, metabolism, redox homeostasis, angiogenesis, and GFR signaling. As seen that insulin-like signaling leads to PI(3)K activation, this tie induces AKT to inhibit FOXO by phosphorylation. Also, the human tumor suppressor PTEN inhibits AKT activity, conceivably by phosphorylation of PIP3. In the setting of oxidative stress, cells with high AMP/ATP ratios determine an increase of JNK and AMPK. Both kinases activate FOXOs by phosphorylation, and the active form of FOXOs relocates to the nucleus. Once in the nucleus, it promotes the expression of genes that promote longevity (75). Oxidative stress activates JNK with the consequence to increase the FOXOs activity. Subsequently, FOXOs reduces both WNT signaling and insulin-like signaling (ILS). The decreased Wnt signaling leads to protein aggregation, which involves the early cell degeneration with the formation of abnormal exocellular bodies, e.g., β-amyloid plaques. The decrease of ILS heads to insulin resistance, hyperglycemia, and development of T2DM (30, 76).

## Osteosarcoma: The Most Frequent Malignant Mesenchymal Tumor of Bone

**Figure 2:**
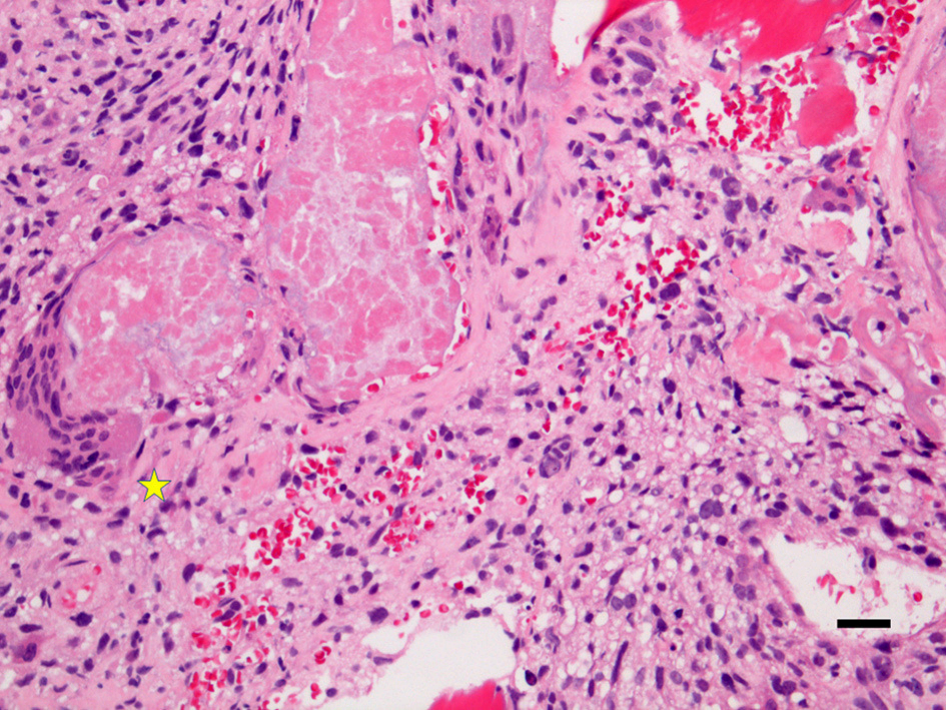
This is an original microphotograph (X200, Hematoxylin & Eosin staining) of osteosarcoma showing osteoclastogenesis (left, asterisk) and osteoblastogenesis (right). The diagnosis of osteosarcoma relies on the identification of anaplastic cells with the osteoid formation (Hematoxylin and Eosin staining, X100 original magnification, bar: 20 μm).

The osteogenic sarcoma of the skeletal system or bone osteosarcoma is a malignant mesenchymal tumor of bone. It is the most common primary bony malignancy with both active osteoclastogenesis and osteoblastogenesis (Figure 2). The osteosarcoma derives from primitive bone-forming mesenchymal cells. The incidence rates of osteosarcoma per year per million of persons are about 4.0% for the range of 0–14 years and 5.0% for children aged 0–19 years, being eighth (2.4%) in the pediatric cancer incidence after leukemia (30%), brain tumors (22.3%), neuroblastoma (7.3%), nephroblastoma (5.6%), lymphoma of non-Hodgkin type (4.5%), rhabdomyosarcoma (3.1%), and retinoblastoma (2.8%) (1). Similar to other neoplasms, osteosarcoma has a bimodal age distribution, having the first peak during adolescence and the second peak in the elderly. Since the first peak is in the 10-14-year-old age group, there is an essential endocrinologic coincidence to be considered. This time corresponds to the pubertal growth spurt and may indicate a close relationship between the endocrinologic changes occurring during the adolescent growth spurt and the endocrinologic platforms of the bone metabolism. In the elderly osteosarcoma, there is a strong link with Paget disease of the bone. That there is a connection with the pubertal growth spurt, it may also be supported from the most frequent site of osteosarcoma. Indeed, it occurs near the metaphyseal growth plates of the long bones of the extremities (1). Although death rates for osteosarcoma have been declining by about 1% per year, the global 5 year survival rate for this tumor is 68%, without substantial gender difference. It has been suggested that osteosarcoma is more often identified in patients with abnormal glucose metabolism, although clear epidemiology data are lacking (77–80). The abnormal glucose metabolism may also be relevant locally other than generally. Interestingly, a teenager was described harboring a premature aging syndrome with diabetes mellitus, osteoporosis, and osteosarcoma (81). By studying animal models, spontaneous osteosarcoma was found in about 7% diabetic (non-obese) mice (79, 80). In osteosarcoma, genetics changes include point-mutations, aneuploidy, and chromothripsis, in which there are numerous rearrangements of the genome. It leads to oscillations of the copy number states, which has been labeled as a dramatic cellular catastrophe (82, 83). There is chromosomal instability in osteogenic osteosarcoma. This instability leads to the breakdown of the cell-cycle checkpoints and DNA-repair mechanisms. Moreover, there are numerous aneuploidy losses or gains at multiple chromosomal sites. Chromosomes 9, 10, 13, and 17 may be lost. The chromosomes 3, 6, 9, 10, 13, 17, and 18 may have a deletion of some parts of the chromosome. Amplification of chromosomal parts can also be detected. DNA sequence copy number upsurges have been distinguished on the regions 1q21, 3q26, 6p, 8q, 12q12-13, 14q24-qter, 17p11-12 of the autosomes and on the regions Xp11.2-21, and Xq12 of the allosomes. In most of the regions harboring chromosomal changes, there are sites of tumor suppressors and oncogenes. Apart of tumor suppressor and oncogenic sites, there are regions involved in the transcription procedure of the genetic information, including c-MYC and c-FOS that seem to play substantial roles in the etiology and/or pathogenesis (84, 85). In listing the other oncogenes connected with amplifications in osteosarcoma, we need to mention Cell Division Cycle five-Like (*CDC5L*), Mitogen-Activated Protein Kinase 7 (*MAPK7*), Mesenchymal to Epithelial Transition (*MET*), *PIM1*, peripheral myelin protein 22 (*PMP22*), DNA Primase Subunit 1 (*PRIM1*) other than Runt Related Transcription Factor 2 (*RUNX2*), and Vascular endothelial growth factor A (*VEGFA*) (86). Of these genes, *MAPK7, MET, PIM1, PMP22, RUNX2*, and *VEGFA* have been described to be associated with diabetes (87–91). Finally, epigenetic changes have also been demonstrated to play a role in osteosarcoma. Epigenetic modifications are specific changes in gene expression that are not due to direct changes in the DNA sequence. In osteosarcoma, there are epigenetic changes, which include methylation of DNA and modification of histones, nucleosome remodeling, and RNA-facilitated events (84, 92–94). Among others, p16 is a tumor-suppressor protein that plays a central role in osteosarcoma. The tumor suppressor p16 is a cell-cycle regulation factor, which acts by decelerating cells progression. The methylation of the cytosine residue within a gene can alter its expression. This event occurs in cytosine-phosphate-guanine (CpG) islands, i.e., DNA sites of 200 bp or more, GC rate >50%, and a detected-to-expected CpG ratio >60%. Gene silencing is the consequence of the methylation of CpG islands in promoter regions. The hypermethylation, when it occurs, reduces the genetic expression at the p16INK4 locus (84). Moreover, lysine-specific demethylase 1 (LSD1), a histone demethylase of the cell, is overexpressed in osteosarcoma. Cell lines treated with the inhibitor of LSD1 exhibit reduced cell growth (84, 95). Likewise, demethylation of the promoter regions of TSSC3, which is a proapoptotic gene, caused in overexpression of the gene with the consequence of suppression of the osteosarcoma cells (96). The demethylation of tumor-suppressor genes in osteosarcoma seems to alter the metastatic capability of the tumor (97, 98). Some microRNAs have also been identified and suggested to be markers of prognosis influencing the genetic expression of osteosarcoma (99). Transcription factors, which enable the process of transcribing coding information from the DNA to single-stranded RNA by binding to promoter sequences on the genome, are carefully supervised in cells at different levels. Osteosarcoma cells show that such regulatory mechanism is disturbed. The study of both FOS and JUN has evidenced that their oncogenes are upregulated in osteosarcoma. Both FOS and JUN are components of the activator protein one complex, which is a transcription factor that controls cell proliferation, differentiation, and metabolism in the bone. The activator protein one complex has also been concerned in the tendency of these tumors to invade and metastasize (100–102). Intranuclear MYC, which is a transcription factor and endorses cellular growth and proliferation, is also overexpressed in osteosarcoma. It is also linked with struggle to conventional chemotherapy protocols (85, 103, 104). That transcription factors play an important role in osteosarcoma may be underlined by the discovery that niclosamide, a traditional anti-helminthic drug, is successful in some osteosarcomas by inducing apoptosis and inhibiting cell-cycle progression in osteosarcoma cells. Niclosamide inhibits the transcription factors E2F1, AP1, and c-MYC-responsive reporters strongly (105, 106). Melatonin, which is a hormone, produced mainly by the pineal gland, regulates wakefulness, and protects mesenchymal stem cells of the bone marrow against iron overload-induced aberrant differentiation and cellular senescence, has also been seen influencing the progression of osteosarcoma *in vitro*. Melatonin weakens osteosarcoma cell invasion by inhibition of the c-Jun N-terminal kinase pathway (107, 108). Melatonin increased and decreased the activation of ERK 1/2 and JNK 1/2, respectively, in a dose-dependent manner in U2OS and HOS osteosarcoma cells (108). This occurred while exerting no apparent influence on the level and activation of P38, AKT, PTK2 protein tyrosine kinase 2 (PTK2), also known as focal adhesion kinase or FAK, steroid receptor coactivator, or Rapidly accelerated fibrosarcoma (RAF).

## IGF-1, SIRT1, and FOXO1 in Osteosarcoma

In osteosarcoma, increased levels of IGF-1 and IGF-1R have been found that seem to lead to tumor progression through transformation, proliferation, decreased susceptibility to apoptosis, and a phenotype prone to metastasis, including increase cell motility, invasion, and angiogenesis (109–111). Also, an over-expression of IGF-1/IGF-1R signaling also contributes to tumor cell survival, metastasis, and resistance to chemotherapy protocols. Some worrisome data for patients harboring osteosarcoma regard the interaction of IGF-1 signaling and osteosarcoma suggesting that supplementation of osteosarcoma cell lines with IGF-1 increases their growth (112, 113). IGF-1 stimulates cell growth, and proliferation exceeds cell death. In fact, the IGF-1 signaling pathway is activated in osteosarcoma promoting proliferation and chemotherapy resistance by activating the AKT signaling pathway. Jentzsch et al.'s study suggest that local IGF-1 expression is associated with more aggressive tumor types. Although relatively rare in human, the osteosarcoma is 27 times more frequent in dogs (114). In the canine osteosarcoma, IGF-1R mRNA and protein expression are strictly associated with surgical stage and distant metastasis (115). MacEwen et al. have indeed demonstrated that IGF-1R expression is correlated with a poor prognosis of both human and canine osteosarcoma (116).

The role of SIRT1 in cancer is controversially discussed. SIRT1 promotes osteosarcoma metastasis by regulating the expression of genes that are associated with a metastatic phenotype. Zhang et al. found that SIRT1 was upregulated in most primary osteosarcoma tumors when compared with normal tissues (117). In this investigation, Zhang et al. hypothesize that SIRT1 might be coupled with a metastatic phenotype in human osteosarcoma. Cell migration and wound-healing assays supported the invasive activity of osteosarcoma cells and downregulating SIRT1 with shRNA inhibition determined that the migration ability of osteosarcoma cells *in vitro* decreased. It blocked tumor lung metastasis in mice (117). Other groups have challenged this data. In other investigations, SIRT1 inhibits tumor progression, deacetylates β-catenin, drives cell proliferation, and inhibits carcinogenesis in patients harboring colon cancer (118, 119). These incongruencies have been explained considering the different features of various tumors.

FOXO1 is generally low or absent in osteosarcoma, and the FOXO1 locus has been associated with copy number variation (CNV) and loss of heterozygosity (LOH) in osteosarcoma confirming that chromosomal aberrations may be (at least partially) responsible for the low FOXO1 expression in some cases of osteosarcoma (120). In osteosarcoma cell lines, the activation of FOXO1 promotes the pathway leading to cell cycle arrest and apoptosis that has been associated with repressed Wnt/β-catenin signaling. The inhibition of FOXO1 induced cell proliferation and decreased the osteogenic differentiation of osteosarcoma cells. By rebuilding the FOXO1 activity, there was impaired proliferation and apoptosis. Both the retinoblastoma-1 gene (RB1) and FOXO1 are situated in 13q14. This locus is often associated with recurrent losses in osteosarcoma (121–123). Interestingly, patients that harbor a loss of 13q14 show lower event-free survival. Other tumors, including cellular angiofibroma, spindle cell lipoma, as well as mammary and vaginal myofibroblastomas, may share a monoallelic loss of RB1 and FOXO1, although none of them is a malignant tumor (124). Guan et al. (120) identified no point mutations in the coding sequence or DNA hypermethylation in the promoter region of FOXO1, which is similar to what has been published in previous studies, i.e., FOXO1 might be involved in the genomic loss, but rarely engaged in mutation or DNA hypermethylation in tumorigenesis. The same authors found five gains, six losses, and 15 cases of LOH of the FOXO1 locus in 34 cases of osteosarcoma by analysis of whole-genome sequencing (120). Guan et al. found that FOXO1 represses the survival of osteosarcoma cells by inhibition of the Wnt/β-catenin signaling, showing that the inhibition of Wnt/β-catenin signaling by FOXOs is conserved during the development of bone and osseous tumorigenesis. FOXO1 does not seem to have any significant influence on the subcellular localization of β-catenin, but FOXO1 inhibits expression of β-catenin. FOXO1 activation induces cell cycle arrest and its inhibition by impairing osteogenic differentiation of osteosarcoma cell lines considering that FOXO1 is a positive promoter of osteoblastogenesis *in vitro* (125). FOXO1 loss might contribute to the disturbed terminal differentiation observed in osteosarcoma. The rebuilding of FOXO1 activity could be a potential therapeutic strategy for therapy of osteosarcoma.

## Conclusions

There is an intricate relationship that does occur in the interaction of IGF1, SIRT1, and FOXOs in the skeletal system. This relationship is particularly essential not only for altered bone metabolism, i.e., deposition and absorption of osteoid but also putatively for the inflammatory status that may trigger osteosarcoma to develop. A research platform in metabolomics of bone tumors is growing, and the mole of data will shape the treatment of osteosarcoma of the 21st century.

## Author Contributions

CS conceived the study and wrote the manuscript. FS critically reviewed the signaling pathways. S-ML critically reviewed the manuscript. All authors approved the final manuscript.

### Conflict of Interest Statement

The authors declare that the research was conducted in the absence of any commercial or financial relationships that could be construed as a potential conflict of interest.

## References

[1] Ottaviani G Jaffe N. The epidemiology of osteosarcoma. Cancer Treat Res. (2009) 152:3–13. 10.1007/978-1-4419-0284-9_1 20213383

[2] Ogura K Higashi T Kawai A Statistics of bone sarcoma in Japan: report from the Bone and soft tissue tumor registry in Japan. J Orthop Sci. (2017) 22:133–43. 10.1016/j.jos.2016.10.006 27847134

[3] Jung M Tuischer JS Sergi C Gotterbarm T Pohl J Richter W. Local application of a collagen type I/hyaluronate matrix and growth and differentiation factor 5 influences the closure of osteochondral defects in a minipig model by enchondral ossification. Growth Factors. (2006) 24:225–32. 10.1080/08977190600926969 17381063

[4] Simank HG Sergi C Jung M Adolf S Eckhardt C Ehemann V. Effects of local application of growth and differentiation factor-5 (GDF-5) in a full-thickness cartilage defect model. Growth Factors. (2004) 22:35–43. 10.1080/08977190310001645207 15176457

[5] Simank HG Herold F Schneider M Maedler U Ries R Sergi C. Growth and differentiation factor 5 (GDF-5) composite improves the healing of necrosis of the femoral head in a sheep model. Analysis of an animal model. Orthopade. (2004) 33:68–75. 10.1007/s00132-003-0541-z 14747913

[6] Manggold J Sergi C Becker K Lukoschek M Simank HG. A new animal model of femoral head necrosis induced by intraosseous injection of ethanol. Lab Anim. (2002) 36:173–80. 10.1258/0023677021912460 11943082

[7] Simank HG Manggold J Sebald W Ries R Richter W Ewerbeck V. Bone morphogenetic protein-2 and growth and differentiation factor-5 enhance the healing of necrotic bone in a sheep model. Growth Factors. (2001) 19:247–57. 10.3109/08977190109001090 11811780

[8] Gottlieb S Ruvkun G. daf-2, daf-16 and daf-23: genetically interacting genes controlling Dauer formation in Caenorhabditis elegans. Genetics. (1994) 137:107–20. 8056303 10.1093/genetics/137.1.107 PMC1205929

[9] Wolkow CA Munoz MJ Riddle DL Ruvkun G. Insulin receptor substrate and p55 orthologous adaptor proteins function in the Caenorhabditis elegans daf-2/insulin-like signaling pathway. J Biol Chem. (2002) 277:49591–7. 10.1074/jbc.M207866200 12393910

[10] Weinkove D Halstead JR Gems D Divecha N. Long-term starvation and ageing induce AGE-1/PI 3-kinase-dependent translocation of DAF-16/FOXO to the cytoplasm. BMC Biol. (2006) 4:1. 10.1186/1741-7007-4-1 16457721 PMC1403811

[11] Tissenbaum HA. DAF-16: FOXO in the Context of *C*. elegans. Curr Top Dev Biol. (2018) 127:1–21. 10.1016/bs.ctdb.2017.11.007 29433733

[12] Sun X Chen WD Wang YD. DAF-16/FOXO transcription factor in aging and longevity. Front Pharmacol. (2017) 8:548. 10.3389/fphar.2017.00548 28878670 PMC5572328

[13] Giatti S Mastrangelo R D'Antonio M Pesaresi M Romano S Diviccaro S. Neuroactive steroids and diabetic complications in the nervous system. Front Neuroendocrinol. (2018) 48:58–69. 10.1016/j.yfrne.2017.07.006 28739507

[14] Gabay O Sanchez C. Epigenetics, sirtuins and osteoarthritis. Joint Bone Spine. (2012) 79:570–3. 10.1016/j.jbspin.2012.04.005 22738809

[15] Gabay O Clouse KA. Epigenetics of cartilage diseases. Joint Bone Spine. (2016) 83:491–4. 10.1016/j.jbspin.2015.10.004 26723856

[16] Wang Y He J Liao M Hu M Li W Ouyang H. An overview of Sirtuins as potential therapeutic target: structure, function and modulators. Eur J Med Chem. (2019) 161:48–77. 10.1016/j.ejmech.2018.10.028 30342425

[17] Zhou S Tang X Chen HZ. Sirtuins and insulin resistance. Front Endocrinol. (2018) 9:748. 10.3389/fendo.2018.00748 30574122 PMC6291425

[18] Song J Yang B Jia X Li M Tan W Ma S Distinctive roles of Sirtuins on diabetes, protective or detrimental? Front Endocrinol. (2018) 9:724 10.3389/fendo.2018.00724 PMC6284472 30559718

[19] Zhu S Dong Z Ke X Hou J Zhao E Zhang K. The roles of sirtuins family in cell metabolism during tumor development. Semin Cancer Biol. (2018). 10.1016/j.semcancer.2018.11.003. [Epub ahead of print]. 30453040

[20] Strømland Ø Niere M Nikiforov AA VanLinden MR Heiland I Ziegler M. Keeping the balance in NAD metabolism. Biochem Soc Trans. (2019). 10.1042/BST20180417. [Epub ahead of print]. 30626706

[21] Morigi M Perico L Benigni A. Sirtuins in renal health and disease. J Am Soc Nephrol. (2018) 29:1799–809. 10.1681/ASN.2017111218 29712732 PMC6050939

[22] Yamagata K Yoshizawa T. Transcriptional regulation of metabolism by SIRT1 and SIRT7. Int Rev Cell Mol Biol. (2018) 335:143–66. 10.1016/bs.ircmb.2017.07.009 29305011

[23] Chen R Xu M Hogg RT Li J Little B Gerard RD. The acetylase/deacetylase couple CREB-binding protein/Sirtuin 1 controls hypoxia-inducible factor 2 signaling. J Biol Chem. (2012) 287:30800–11. 10.1074/jbc.M111.244780 22807441 PMC3436323

[24] Abed E Delalandre A Lajeunesse D. Beneficial effect of resveratrol on phenotypic features and activity of osteoarthritic osteoblasts. Arthritis Res Ther. (2017) 19:151. 10.1186/s13075-017-1365-2 28666466 PMC5493084

[25] Tamaki N Cristina Orihuela-Campos R Inagaki Y Fukui M Nagata T Ito HO. Resveratrol improves oxidative stress and prevents the progression of periodontitis via the activation of the Sirt1/AMPK and the Nrf2/antioxidant defense pathways in a rat periodontitis model. Free Radic Biol Med. (2014) 75:222–9. 10.1016/j.freeradbiomed.2014.07.034 25091897

[26] Huang W Shang WL Wang HD Wu WW Hou SX. Sirt1 overexpression protects murine osteoblasts against TNF-alpha-induced injury in vitro by suppressing the NF-kappaB signaling pathway. Acta Pharmacol Sin. (2012) 33:668–74. 10.1038/aps.2011.189 22447223 PMC4010359

[27] Jeong WY Kim JB Kim HJ Kim CW. Extremely low-frequency electromagnetic field promotes astrocytic differentiation of human bone marrow mesenchymal stem cells by modulating SIRT1 expression. Biosci Biotechnol Biochem. (2017) 81:1356–62. 10.1080/09168451.2017.1308243 28351214

[28] Magni M Buscemi G Zannini L. Cell cycle and apoptosis regulator 2 at the interface between DNA damage response and cell physiology. Mutat Res. (2018) 776:1–9. 10.1016/j.mrrev.2018.03.004 29807573

[29] Beyfuss K Hood DA. A systematic review of p53 regulation of oxidative stress in skeletal muscle. Redox Rep. (2018) 23:100–17. 10.1080/13510002.2017.1416773 29298131 PMC6748683

[30] Erol A. Insulin resistance is an evolutionarily conserved physiological mechanism at the cellular level for protection against increased oxidative stress. Bioessays. (2007) 29:811–8. 10.1002/bies.20618 17621670

[31] Liu G Bi Y Xue L Zhang Y Yang H Chen X. Dendritic cell SIRT1-HIF1alpha axis programs the differentiation of CD4+ T cells through IL-12 and TGF-beta1. Proc Natl Acad Sci USA. (2015) 112:E957–65. 10.1073/pnas.1420419112 25730867 PMC4352801

[32] Lo Sasso G Menzies KJ Mottis A Piersigilli A Perino A Yamamoto H. SIRT2 deficiency modulates macrophage polarization and susceptibility to experimental colitis. PLoS ONE. (2014) 9:e103573. 10.1371/journal.pone.0103573 25072851 PMC4114785

[33] Ning Z Li Y Liu D Owoicho Orgah J Zhu J Wang Y. Tetrahydroxystilbene glucoside delayed senile symptoms in old mice via regulation of the AMPK/SIRT1/PGC-1alpha signaling cascade. Gerontology. (2018) 64:457–65. 10.1159/000487360 29804119

[34] Fujita Y Yamashita T. Sirtuins in neuroendocrine regulation and neurological diseases. Front Neurosci. (2018) 12:778. 10.3389/fnins.2018.00778 30416425 PMC6213750

[35] Hsu YC Wu YT Tsai CL Wei YH. Current understanding and future perspectives of the roles of sirtuins in the reprogramming and differentiation of pluripotent stem cells. Exp Biol Med. (2018) 243:563–75. 10.1177/1535370218759636 29557214 PMC5882022

[36] Karbasforooshan H Roohbakhsh A Karimi G. SIRT1 and microRNAs: the role in breast, lung and prostate cancers. Exp Cell Res. (2018) 367:1–6. 10.1016/j.yexcr.2018.03.023 29574020

[37] Jiang W Zhang X Hao J Shen J Fang J Dong W. SIRT1 protects against apoptosis by promoting autophagy in degenerative human disc nucleus pulposus cells. Sci Rep. (2014) 4:7456. 10.1038/srep07456 25503852 PMC4264007

[38] Girardelli M Basaldella F Paolera SD Vuch J Tommasini A Martelossi S. Genetic profile of patients with early onset inflammatory bowel disease. Gene. (2018) 645:18–29. 10.1016/j.gene.2017.12.029 29248579

[39] Chiu B Jantuan E Shen F Chiu B Sergi C. Autophagy-inflammasome interplay in heart failure: a systematic review on basics, pathways, and therapeutic perspectives. Ann Clin Lab Sci. (2017) 47:243–52. 28667023

[40] Truong VL Jun M Jeong WS. Role of resveratrol in regulation of cellular defense systems against oxidative stress. Biofactors. (2018) 44:36–49. 10.1002/biof.1399 29193412

[41] Yoon DS Choi Y Lee JW. Cellular localization of NRF2 determines the self-renewal and osteogenic differentiation potential of human MSCs via the P53-SIRT1 axis. Cell Death Dis. (2016) 7:e2093. 10.1038/cddis.2016.3 26866273 PMC4849161

[42] Xin Z Ma Z Hu W Jiang S Yang Z Li T. FOXO1/3: Potential suppressors of fibrosis. Ageing Res Rev. (2018) 41:42–52. 10.1016/j.arr.2017.11.002 29138094

[43] Luo CT Li MO. Foxo transcription factors in T cell biology and tumor immunity. Semin Cancer Biol. (2018) 50:13–20. 10.1016/j.semcancer.2018.04.006 29684436 PMC5986613

[44] Tia N Singh AK Pandey P Azad CS Chaudhary P Gambhir IS. Role of Forkhead Box O (FOXO) transcription factor in aging and diseases. Gene. (2018) 648:97–105. 10.1016/j.gene.2018.01.051 29428128

[45] Ma J Matkar S He X Hua X. FOXO family in regulating cancer and metabolism. Semin Cancer Biol. (2018) 50:32–41. 10.1016/j.semcancer.2018.01.018 29410116

[46] Monsalve M Prieto I de Bem AF Olmos Y. Methodological Approach for the Evaluation of FOXO as a positive regulator of antioxidant genes. Methods Mol Biol. (2019) 1890:61–76. 10.1007/978-1-4939-8900-3_6 30414145

[47] Mukherjee A Hollern DP Williams OG Rayburn TS Byrd WA Yates C. A Review of FOXI3 regulation of development and possible roles in cancer progression and metastasis. Front Cell Dev Biol. (2018) 6:69. 10.3389/fcell.2018.00069 30018953 PMC6038025

[48] Kim HN Iyer S Ring R Almeida M. The role of FoxOs in bone health and disease. Curr Top Dev Biol. (2018) 127:149–63. 10.1016/bs.ctdb.2017.10.004 29433736

[49] Jiang S Li T Yang Z Hu W Yang Y Deciphering the roles of FOXO1 in human neoplasms. Int J Cancer. (2018) 143:1560–8. 10.1002/ijc.31338 29473160

[50] Miao C Li Y Zhang X. The functions of FoxO transcription factors in epithelial wound healing. Australas J Dermatol. (2018). 10.1111/ajd.12952. [Epub ahead of print]. 30450624

[51] Yadav RK Chauhan AS Zhuang L Gan B. FoxO transcription factors in cancer metabolism. Semin Cancer Biol. (2018) 50:65–76. 10.1016/j.semcancer.2018.01.004 29309929 PMC5986595

[52] Hou T Li Z Zhao Y Zhu WG. Mechanisms controlling the anti-neoplastic functions of FoxO proteins. Semin Cancer Biol. (2018) 50:101–14. 10.1016/j.semcancer.2017.11.007 29155239

[53] Wang Y Zhou Y Graves DT. FOXO transcription factors: their clinical significance and regulation. Biomed Res Int. (2014) 2014:925350. 10.1155/2014/925350 24864265 PMC4016844

[54] Al-Bahrani R Abuetabh Y Zeitouni N Sergi C. Cholangiocarcinoma: risk factors, environmental influences and oncogenesis. Ann Clin Lab Sci. (2013) 43:195–210. 23694797

[55] Teitelbaum SL Ross FP. Genetic regulation of osteoclast development and function. Nat Rev Genet. (2003) 4:638–49. 10.1038/nrg1122 12897775

[56] Baruzzi A Berton G. The tyrosine kinase Abl is a component of macrophage podosomes and is required for podosome formation and function. Eur J Immunol. (2012) 42:2720–6. 10.1002/eji.201142270 22733220

[57] Ray BJ Thomas K Huang CS Gutknecht MF Botchwey EA Bouton AH. Regulation of osteoclast structure and function by FAK family kinases. J Leukoc Biol. (2012) 92:1021–8. 10.1189/jlb.0512259 22941736 PMC3476245

[58] Smith H Whittall C Weksler B Middleton J. Chemokines stimulate bidirectional migration of human mesenchymal stem cells across bone marrow endothelial cells. Stem Cells Dev. (2012) 21:476–86. 10.1089/scd.2011.0025 21513440

[59] Iyer S Ambrogini E Bartell SM Han L Roberson PK de Cabo R. FOXOs attenuate bone formation by suppressing Wnt signaling. J Clin Invest. (2013) 123:3409–19. 10.1172/JCI68049 23867625 PMC3726166

[60] Iyer S Han L Bartell SM Kim HN Gubrij I de Cabo R. Sirtuin1 (Sirt1) promotes cortical bone formation by preventing beta-catenin sequestration by FoxO transcription factors in osteoblast progenitors. J Biol Chem. (2014) 289:24069–78. 10.1074/jbc.M114.561803 25002589 PMC4148840

[61] Lee YM Shin SI Shin KS Lee YR Park BH Kim EC. The role of sirtuin 1 in osteoblastic differentiation in human periodontal ligament cells. J Periodontal Res. (2011) 46:712–21. 10.1111/j.1600-0765.2011.01394.x 21745208

[62] Piemontese M Onal M Xiong J Han L Thostenson JD Almeida M. Low bone mass and changes in the osteocyte network in mice lacking autophagy in the osteoblast lineage. Sci Rep. (2016) 6:24262. 10.1038/srep24262 27064143 PMC4827128

[63] Baron R Kneissel M. WNT signaling in bone homeostasis and disease: from human mutations to treatments. Nat Med. (2013) 19:179–92. 10.1038/nm.3074 23389618

[64] Clevers H Nusse R. Wnt/beta-catenin signaling and disease. Cell. (2012) 149:1192–205. 10.1016/j.cell.2012.05.012 22682243

[65] Jilka RL O'Brien CA. The role of osteocytes in age-related bone loss. Curr Osteoporos Rep. (2016) 14:16–25. 10.1007/s11914-016-0297-0 26909563

[66] Bartell SM Kim HN Ambrogini E Han L Iyer S Serra Ucer S. FoxO proteins restrain osteoclastogenesis and bone resorption by attenuating H2O2 accumulation. Nat Commun. (2014) 5:3773. 10.1038/ncomms4773 24781012 PMC4015330

[67] Liu W Wang S Wei S Sun L Feng X. Receptor activator of NF-kappaB (RANK) cytoplasmic motif, 369PFQEP373, plays a predominant role in osteoclast survival in part by activating Akt/PKB and its downstream effector AFX/FOXO4. J Biol Chem. (2005) 280:43064–72. 10.1074/jbc.M509006200 16260781

[68] Tan P Guan H Xie L Mi B Fang Z Li J. FOXO1 inhibits osteoclastogenesis partially by antagnozing MYC. Sci Rep. (2015) 5:16835. 10.1038/srep16835 26568463 PMC4645183

[69] Tan T Wang L Wang B Collagen and prostaglandin E(2) regulate aromatase expression through the PI3K/AKT/IKK and the MAP kinase pathways in adipose stromal cells. Mol Med Rep. (2015) 12:4766–72. 10.3892/mmr.2015.3901 26059638

[70] Jais A Einwallner E Sharif O Gossens K Lu TT Soyal SM. Heme oxygenase-1 drives metaflammation and insulin resistance in mouse and man. Cell. (2014) 158:25–40. 10.1016/j.cell.2014.04.043 24995976 PMC5749244

[71] Ambrogini E Almeida M Martin-Millan M Paik JH Depinho RA Han L. FoxO-mediated defense against oxidative stress in osteoblasts is indispensable for skeletal homeostasis in mice. Cell Metab. (2010) 11:136–46. 10.1016/j.cmet.2009.12.009 20142101 PMC2819984

[72] Rached MT Kode A Xu L Yoshikawa Y Paik JH Depinho RA. FoxO1 is a positive regulator of bone formation by favoring protein synthesis and resistance to oxidative stress in osteoblasts. Cell Metab. (2010) 11:147–60. 10.1016/j.cmet.2010.01.001 20142102 PMC2820405

[73] Ferron M Wei J Yoshizawa T Del Fattore A DePinho RA Teti A. Insulin signaling in osteoblasts integrates bone remodeling and energy metabolism. Cell. (2010) 142:296–308. 10.1016/j.cell.2010.06.003 20655470 PMC2910411

[74] Wei H Yu X. Functions of PARylation in DNA Damage Repair Pathways. Genomics Proteomics Bioinformatics. (2016) 14:131–9. 10.1016/j.gpb.2016.05.001 27240471 PMC4936651

[75] Henderson ST Johnson TE. daf-16 integrates developmental and environmental inputs to mediate aging in the nematode Caenorhabditis elegans. Curr Biol. (2001) 11:1975–80. 10.1016/S0960-9822(01)00594-2 11747825

[76] Manolopoulos KN Klotz LO Korsten P Bornstein SR Barthel A. Linking Alzheimer's disease to insulin resistance: the FoxO response to oxidative stress. Mol Psychiatry. (2010) 15:1046–52. 10.1038/mp.2010.17 20966918

[77] Mishra PJ. MicroRNAs as promising biomarkers in cancer diagnostics. Biomark Res. (2014) 2:19. 10.1186/2050-7771-2-19 25356314 PMC4212235

[78] Schmid C Ghirlanda-Keller C Zapf J. Effects of IGF-I and -II, IGF binding protein-3 (IGFBP-3), and transforming growth factor-beta (TGF-beta) on growth and apoptosis of human osteosarcoma Saos-2/B-10 cells: lack of IGF-independent IGFBP-3 effects. Eur J Endocrinol. (2001) 145:213–21. 11454519 10.1530/eje.0.1450213

[79] Gilbert MP Pratley RE. The impact of diabetes and diabetes medications on bone health. Endocr Rev. (2015) 36:194–213. 10.1210/er.2012-1042 25738213

[80] Kavirayani AM Foreman O. Retrospective study of spontaneous osteosarcomas in the nonobese diabetic strain and nonobese diabetic-derived substrains of mice. Vet Pathol. (2010) 47:482–7. 10.1177/0300985810363699 20348488

[81] Okamoto N Satomura K Hatsukawa Y Hayashida M Saijo K Ohno T. Premature aging syndrome with osteosarcoma, cataracts, diabetes mellitus, osteoporosis, erythroid macrocytosis, severe growth and developmental deficiency. Am J Med Genet. (1997) 69:169–70. 9056555

[82] Stephens PJ Greenman CD Fu B Yang F Bignell GR Mudie LJ. Massive genomic rearrangement acquired in a single catastrophic event during cancer development. Cell. (2011) 144:27–40. 10.1016/j.cell.2010.11.055 21215367 PMC3065307

[83] Maher CA Wilson RK. Chromothripsis and human disease: piecing together the shattering process. Cell. (2012) 148:29–32. 10.1016/j.cell.2012.01.006 22265399 PMC3658123

[84] Morrow JJ Khanna C. Osteosarcoma genetics and epigenetics: emerging biology and candidate therapies. Crit Rev Oncogenesis. (2015) 20:173–97. 10.1615/CritRevOncog.2015013713 26349415 PMC4894524

[85] Gamberi G Benassi MS Bohling T Ragazzini P Molendini L Sollazzo MR. C-myc and c-fos in human osteosarcoma: prognostic value of mRNA and protein expression. Oncology. (1998) 55:556–63. 9778623 10.1159/000011912

[86] Mutsaers AJ Walkley CR Cells of origin in osteosarcoma: mesenchymal stem cells or osteoblast committed cells? Bone. (2014) 62:56–63. 10.1016/j.bone.2014.02.003 24530473

[87] Zhu Y Ma WQ Han XQ Wang Y Wang X Liu NF. Advanced glycation end products accelerate calcification in VSMCs through HIF-1alpha/PDK4 activation and suppress glucose metabolism. Sci Rep. (2018) 8:13730. 10.1038/s41598-018-31877-6 30213959 PMC6137084

[88] Zhang YY Li C Yao GF Du LJ Liu Y Zheng XJ. Deletion of macrophage mineralocorticoid receptor protects hepatic steatosis and insulin resistance through ERalpha/HGF/Met pathway. Diabetes. (2017) 66:1535–47. 10.2337/db16-1354 28325853 PMC5860190

[89] Katare R Caporali A Zentilin L Avolio E Sala-Newby G Oikawa A. Intravenous gene therapy with PIM-1 via a cardiotropic viral vector halts the progression of diabetic cardiomyopathy through promotion of prosurvival signaling. Circ Res. (2011) 108:1238–51. 10.1161/CIRCRESAHA.110.239111 21474815

[90] Lomas-Soria C Perez-Ramirez IF Caballero-Perez J Guevara-Gonzalez RG Guevara-Olvera L Loarca-Pina G. Cooked common beans (Phaseolus vulgaris L.) modulate renal genes in streptozotocin-induced diabetic rats. J Nutr Biochem. (2015) 26:761–8. 10.1016/j.jnutbio.2015.02.006 25863648

[91] Sun AP Tang L Liao Q Zhang H Zhang YS Zhang J. Coexistent charcot-marie-tooth type 1A and type 2 diabetes mellitus neuropathies in a Chinese family. Neural Regen Res. (2015) 10:1696–9. 10.4103/1673-5374.167771 26692872 PMC4660768

[92] Li B Ye Z. Epigenetic alterations in osteosarcoma: promising targets. Mol Biol Rep. (2014) 41:3303–15. 10.1007/s11033-014-3193-7 24500341

[93] Roberts SB Wootton E De Ferrari L Albagha OM Salter DM. Epigenetics of osteoarticular diseases: recent developments. Rheumatol Int. (2015) 35:1293–305. 10.1007/s00296-015-3260-y 25812537

[94] Cui J Wang W Li Z Zhang Z Wu B Zeng L. Epigenetic changes in osteosarcoma. Bull Cancer. (2011) 98:E62–8. 10.1684/bdc.2011.1400 21708514

[95] Bennani-Baiti IM Machado I Llombart-Bosch A Kovar H. Lysine-specific demethylase 1 (LSD1/KDM1A/AOF2/BHC110) is expressed and is an epigenetic drug target in chondrosarcoma, Ewing's sarcoma, osteosarcoma, and rhabdomyosarcoma. Hum Pathol. (2012) 43:1300–7. 10.1016/j.humpath.2011.10.010 22245111

[96] Li Y Huang Y Lv Y Meng G Guo QN. Epigenetic regulation of the pro-apoptosis gene TSSC3 in human osteosarcoma cells. Biomed Pharmacother. (2014) 68:45–50. 10.1016/j.biopha.2013.10.006 24268429

[97] Mu X Sultankulov B Agarwal R Mahjoub A Schott T Greco N. Chick embryo extract demethylates tumor suppressor genes in osteosarcoma cells. Clin Orthopaedics Relat Res. (2014) 472:865–73. 10.1007/s11999-013-3104-6 23761177 PMC3916611

[98] Zhou C Tan W Lv H Gao F Sun J. Hypoxia-inducible microRNA-488 regulates apoptosis by targeting Bim in osteosarcoma. Cell Oncol. (2016) 39:463–71. 10.1007/s13402-016-0288-2 27376839 PMC13001872

[99] Dong J Liu Y Liao W Liu R Shi P Wang L. miRNA-223 is a potential diagnostic and prognostic marker for osteosarcoma. J Bone Oncol. (2016) 5:74–9. 10.1016/j.jbo.2016.05.001 27335775 PMC4908189

[100] Broadhead ML Clark JC Myers DE Dass CR Choong PF. The molecular pathogenesis of osteosarcoma: a review. Sarcoma. (2011) 2011:959248. 10.1155/2011/959248 21559216 PMC3087974

[101] Wu JX Carpenter PM Gresens C Keh R Niman H Morris JW. The proto-oncogene c-fos is over-expressed in the majority of human osteosarcomas. Oncogene. (1990) 5:989–1000. 2115647

[102] Franchi A Calzolari A Zampi G. Immunohistochemical detection of c-fos and c-jun expression in osseous and cartilaginous tumours of the skeleton. Virchows Archiv Int J Pathol. (1998) 432:515–9. 9672192 10.1007/s004280050199

[103] Shimizu T Ishikawa T Sugihara E Kuninaka S Miyamoto T Mabuchi Y. c-MYC overexpression with loss of Ink4a/Arf transforms bone marrow stromal cells into osteosarcoma accompanied by loss of adipogenesis. Oncogene. (2010) 29:5687–99. 10.1038/onc.2010.312 20676132

[104] Scionti I Michelacci F Pasello M Hattinger CM Alberghini M Manara MC. Clinical impact of the methotrexate resistance-associated genes C-MYC and dihydrofolate reductase (DHFR) in high-grade osteosarcoma. Ann Oncol. (2008) 19:1500–8. 10.1093/annonc/mdn148 18385200

[105] Liao Z Nan G Yan Z Zeng L Deng Y Ye J. The anthelmintic drug niclosamide inhibits the proliferative activity of human osteosarcoma cells by targeting multiple signal pathways. Curr Cancer Drug Targets. (2015) 15:726–38. 10.2174/1568009615666150629132157 26118906

[106] Osasan S Zhang M Shen F Paul PJ Persad S Sergi C. Osteogenic Sarcoma: A 21st century review. Anticancer Res. (2016) 36:4391–8. 10.21873/anticanres.10982 27630274

[107] Yang F Yang L Li Y Yan G Feng C Liu T. Melatonin protects bone marrow mesenchymal stem cells against iron overload-induced aberrant differentiation and senescence. J Pineal Res. (2017) 63:e12422. 10.1111/jpi.12422 28500782

[108] Lu KH Su SC Lin CW Hsieh YH Lin YC Chien MH. Melatonin attenuates osteosarcoma cell invasion by suppression of C-C motif chemokine ligand 24 through inhibition of the c-Jun N-terminal kinase pathway. J Pineal Res. (2018) 65:e12507. 10.1111/jpi.12507 29766567

[109] Hong SH Briggs J Newman R Hoffman K Mendoza A LeRoith D. Murine osteosarcoma primary tumour growth and metastatic progression is maintained after marked suppression of serum insulin-like growth factor I. Int J Cancer. (2009) 124:2042–9. 10.1002/ijc.24169 19132750 PMC6948843

[110] Jentzsch T Robl B Husmann M Bode-Lesniewska B Fuchs B. Worse prognosis of osteosarcoma patients expressing IGF-1 on a tissue microarray. Anticancer Res. (2014) 34:3881–9. 25075009

[111] Viereck V Siggelkow H Pannem R Braulke T Scharf JG Kubler B. Alteration of the insulin-like growth factor axis during *in vitro* differentiation of the human osteosarcoma cell line HOS 58. J Cell Biochem. (2007) 102:28–40. 10.1002/jcb.21274 17372931

[112] Wang YH Han XD Qiu Y Xiong J Yu Y Wang B. Increased expression of insulin-like growth factor-1 receptor is correlated with tumor metastasis and prognosis in patients with osteosarcoma. J Surg Oncol. (2012) 105:235–43. 10.1002/jso.22077 21866554

[113] Borinstein SC Barkauskas DA Bernstein M Goorin A Gorlick R Krailo M. Analysis of serum insulin growth factor-1 concentrations in localized osteosarcoma: a children's oncology group study. Pediatr Blood Cancer. (2014) 61:749–52. 10.1002/pbc.24778 24178953 PMC3946315

[114] Simpson S Dunning MD de Brot S Grau-Roma L Mongan NP Rutland CS. Comparative review of human and canine osteosarcoma: morphology, epidemiology, prognosis, treatment and genetics. Acta Vet Scand. (2017) 59:71. 10.1186/s13028-017-0341-9 29065898 PMC5655853

[115] Maniscalco L Iussich S Morello E Martano M Gattino F Miretti S. Increased expression of insulin-like growth factor-1 receptor is correlated with worse survival in canine appendicular osteosarcoma. Vet J. (2015) 205:272–80. 10.1016/j.tvjl.2014.09.005 25257352

[116] MacEwen EG Pastor J Kutzke J Tsan R Kurzman ID Thamm DH. IGF-1 receptor contributes to the malignant phenotype in human and canine osteosarcoma. J Cell Biochem. (2004) 92:77-91. 10.1002/jcb.20046 15095405

[117] Zhang N Xie T Xian M Wang YJ Li HY Ying MD. SIRT1 promotes metastasis of human osteosarcoma cells. Oncotarget. (2016) 7:79654–69. 10.18632/oncotarget.12916 27793039 PMC5346743

[118] Firestein R Blander G Michan S Oberdoerffer P Ogino S Campbell J. The SIRT1 deacetylase suppresses intestinal tumorigenesis and colon cancer growth. PLoS ONE. (2008) 3:e2020. 10.1371/journal.pone.0002020 18414679 PMC2289879

[119] Kabra N Li Z Chen L Li B Zhang X Wang C. SirT1 is an inhibitor of proliferation and tumor formation in colon cancer. J Biol Chem. (2009) 284:18210–7. 10.1074/jbc.M109.000034 19433578 PMC2709385

[120] Guan H Tan P Xie L Mi B Fang Z Li J. FOXO1 inhibits osteosarcoma oncogenesis via Wnt/beta-catenin pathway suppression. Oncogenesis. (2015) 4:e166. 10.1038/oncsis.2015.25 26344693 PMC4767937

[121] Kresse SH Ohnstad HO Paulsen EB Bjerkehagen B Szuhai K Serra M. LSAMP, a novel candidate tumor suppressor gene in human osteosarcomas, identified by array comparative genomic hybridization. Genes Chromosomes Cancer. (2009) 48:679–93. 10.1002/gcc.20675 19441093

[122] Blattmann C Thiemann M Stenzinger A Roth EK Dittmar A Witt H. Establishment of a patient-derived orthotopic osteosarcoma mouse model. J Transl Med. (2015) 13:136. 10.1186/s12967-015-0497-x 25926029 PMC4428092

[123] Sadikovic B Park PC Selvarajah S Zielenska M. Array comparative genomic hybridization in osteosarcoma. Methods Mol Biol. (2013) 973:227–47. 10.1007/978-1-62703-281-0_15 23412794

[124] Magro G Righi A Casorzo L Antonietta T Salvatorelli L Kacerovska D. Mammary and vaginal myofibroblastomas are genetically related lesions: fluorescence in situ hybridization analysis shows deletion of 13q14 region. Hum Pathol. (2012) 43:1887–93. 10.1016/j.humpath.2012.01.015 22575260

[125] Siqueira MF Flowers S Bhattacharya R Faibish D Behl Y Kotton DN. FOXO1 modulates osteoblast differentiation. Bone. (2011) 48:1043–51. 10.1016/j.bone.2011.01.019 21281751 PMC3109483

